# Supplementary material for: Conversion of an agonistic anti-TNFR2 biparatopic antibody into an antagonist by insertion of peptide linkers into the hinge region
Source: J Biol Chem. 2025 Jul 31;301(9):110548. doi: 10.1016/j.jbc.2025.110548 (PMC12452656; doi:10.1016/j.jbc.2025.110548)
Supplement: Supplementary Material [file mmc1.pdf]

Supporting Information for

Conversion of an agonistic anti-TNFR2 biparatopic antibody into an antagonist by insertion of peptide linkers into the hinge region

Takuya Otsuki[1], Shigeyuki Matsumoto[2], Junso Fujita[3,4,5], Tomoko Miyata[3,4], Keiichi Namba[3,4], Ryo Kanada[6], Yasushi Okuno[2,6], Haruhiko Kamada[1,7], Hiroaki Ohno[1,7*] and Hiroki Akiba[1,7*]

[1] Graduate School of Pharmaceutical Sciences, Kyoto University, Sakyo-ku, Kyoto 606-8501, Japan

[2] Graduate School of Medicine, Kyoto University, Sakyo-ku, Kyoto 606-8501, Japan

[3] Graduate School of Pharmaceutical Sciences, The University of Osaka, Suita, Osaka 565-0871, Japan

[4] JEOL YOKOGUSHI Research Alliance Laboratories, The University of Osaka, Suita, Osaka 565-0871, Japan

[5] Graduate School of Pharmaceutical Sciences, The University of Osaka, Suita, Osaka 565-0871, Japan

[6] HPC- and AI-driven Drug Development Platform Division, RIKEN Center for Computational Science, Chuo-ku, Kobe, Hyogo 650-0047, Japan

[7] National Institutes of Biomedical Innovation, Health and Nutrition, Ibaraki, Osaka 567-0085, Japan

*Corresponding authors, hakiba@pharm.kyoto-u.ac.jp (H.A.), hohno@pharm.kyoto-u.ac.jp (H.O.)

Table of Contents

Contents	Title	Page #
Supplementary Table 1	Amino acid sequence of BA1	S4
Supplementary Table 2	Amino acid sequence of BA2	S5
Supplementary Table 3	Amino acid sequence of BA3	S6
Supplementary Table 4	Cryo-EM data collection and processing	S7
Supplementary Table 5	Eigenvalues and contribution of 1st-5th Principal Components (PC1-5)	S7
Supplementary Figure 1	Format exchange from intein-mediated protein trans-splicing (IMPTS) into CrossMAb	S8
Supplementary Figure 2	Preparative size-exclusion chromatograms of BA1 inserted with a peptide linker into one of heavy chains	S9
Supplementary Figure 3	Preparative size-exclusion chromatograms of BA2 inserted with a peptide linker into one of heavy chains	S10
Supplementary Figure 4	Preparative size-exclusion chromatograms of BA3 inserted with a peptide linker into one of heavy chains	S11
Supplementary Figure 5	SDS-PAGE analysis of BpAbs	S12
Supplementary Figure 6	Decomposition of proteins stored at 4 °C, one month after purification	S13
Supplementary Figure 7	Control of biological activities through linker insertion into the hinge region of one of heavy chains	S14
Supplementary Figure 8	Preparative size-exclusion chromatograms of BA1-WT, BA1-GP2, BA1-GP4 and BA1-GP8	S15
Supplementary Figure 9	Preparative cation-exchange chromatograms of BA1-WT, BA1-GP2, BA1-GP4 and BA1-GP8	S16
Supplementary Figure 10	Preparative size-exclusion chromatograms of BA2-WT, BA2-GP2, BA2-GP4 and BA2-GP8	S17
Supplementary Figure 11	Preparative cation-exchange chromatograms of BA2-WT, BA2-GP2, BA2-GP4 and BA2-GP8	S18
Supplementary Figure 12	Agonistic and antagonistic activities of the mixtures of three combinations of two Fab in a reporter gene assay	S19
Supplementary Figure 13	Preparative size-exclusion chromatograms of BA3-WT, BA3-GP2 and BA3-GP4	S20
Supplementary Figure 14	Preparative cation-exchange chromatograms of BA2-GP2 and BA2-GP4	S21

Supplementary Figure 15	Agonistic and antagonistic activities of BA3-WT, BA3 GP2 and BA3-GP4 in a reporter gene assay	S21
Supplementary Figure 16	Preparative size-exclusion and cation-exchange chromatograms of BA1-GP2, BA1-GP4, BA2-GP2 and BA2-GP4 after deglycosylation	S22
Supplementary Figure 17	Agonistic and antagonistic activities of BpAbs after deglycosylation in a reporter gene assay.	S23
Supplementary Figure 18	Apparent binding affinity of BpAbs to TNFR2-expressing Ramos-Blue cells analyzed by using flow cytometry	S23
Supplementary Figure 19	Immunocomplex formed between BA3 and TNFR2	S24
Supplementary Figure 20	Sample preparation for cryo-electron microscopy	S24
Supplementary Figure 21	Image processing of BA1-GP4/TNFR2-MBP complex	S25
Supplementary Figure 22	Amino-acid residues of TNFR2 in the interface with 92-Fab from two different BpAbs	S26
Supplementary Figure 23	The epitope of 96-Fab binding to TNFR2	S27
Supplementary Figure 24	Strategy for building the initial structure of the complexes of BA1-GP2 and TNFR2	S28
Supplementary Figure 25	Strategy for building the initial structure of the complexes of BA1-GP4 and TNFR2	S29
Supplementary Figure 26	Strategy for building ternary complex of 109-Fab, 96-Fab and TNFR2	S30
Supplementary Figure 27	Strategy for building the initial structure of the complexes of BA2-GP2 and TNFR2	S31
Supplementary Figure 28	Strategy for building the initial structure of the complexes of BA2-GP4 and TNFR2	S32

Supplementary Table 1. Amino acid sequence of BA1[a]

Knob chain	Hole chain
HC	***HC***
KVQLQQSGAELVKPGASVKLSCKASGYTFTESIIHWVKQRSGQGLEWIGW<br>FYPGSDNINYNEKFKDKATLTADKSSSTVYMELTRLTSEDSAVYFCASHE<br>GPYVYFDYWGQGTTLTVSSASTKGPSVFPLAPSSKSTSGGTAALGCLVKD<br>YFPEPVTVSWNSGALTSGVHTFPAVLQSSGLYSLSSVVTVPSSSLGTQTY<br>ICNVNHKPSNTKVDKKVEPKS**---Hinge region---**PAPELLGGPSV<br>FLFPPKPKDTLMISRTPEVTCVVVDVSHEDPEVKFNWYVDGVEVHNAKTK<br>PREEQYNSTYRVVSVLTVLHQDWLNGKEYKCKVSNKALPAPIEKTISKAK<br>GQPREPQVYTLPPCRDELTKNQVSLWCLVKGFYPSDIAVEWESNGQPENN<br>YKTTPPVLDSDGSFFLYSKLTVDKSRWQQGNVFSCSVMHEALHNHYTQKS<br>LSLSPGK	EVQLQQSGAELVKPGASVKLSCTPSGFNIKDTYMHWVKQRPEQGLEWIGR<br>IDPANGYTEYDPKFQDKATITADTSSNTAYLQLSSLTSEDTAVYYCADTQ<br>LYYWGQGTTLTVSSASVAAPSVFIFPPSDEQLKSGTASVVCLLNNFYPRE<br>AKVQWKVDNALQSGNSQESVTEQDSKDSTYSLSSTLTLSKADYEKHKVYA<br>CEVTHQGLSSPVTKSFNRGE**---Hinge region---**PAPELLGGPSVF<br>LFPPKPKDTLMISRTPEVTCVVVDVSHEDPEVKFNWYVDGVEVHNAKTKP<br>REEQYNSTYRVVSVLTVLHQDWLNGKEYKCKVSNKALPAPIEKTISKAKG<br>QPREPQVCTLPPSRDELTKNQVSLSCAVKGFYPSDIAVEWESNGQPENNY<br>KTTPPVLDSDGSFFLVSKLTVDKSRWQQGNVFSCSVMHEALHNHYTQKSL<br>SLSPGK
LC	***LC***
DIVMTQSHKFMSTSVGDRVSITCKASQDVSTAVAWYQQKPGQSPKLLIYW<br>TSTRHTGVPDRFTGSGSGTDYTLTISSVQAEDLALYYCQHHYSTPYTFGG<br>GTKLEIQRTVAAPSVFIFPPSDEQLKSGTASVVCLLNNFYPREAKVQWKV<br>DNALQSGNSQESVTEQDSKDSTYSLSSTLTLSKADYEKHKVYACEVTHQG<br>LSSPVTKSFNRGEC	QIVLTQSPAIMSASLGERVTMTCTASSSVSSTYLHWYQQKPGSSPKLWIY<br>STSNLASGVPARFSGSGSGTSYSLTISNMEAEDAATYYCHQYHRSPLTFG<br>AGTKLELKSSASTKGPSVFPLAPSSKSTSGGTAALGCLVKDYFPEPVTVS<br>WNSGALTSGVHTFPAVLQSSGLYSLSSVVTVPSSSLGTQTYICNVNHKPS<br>NTKVDKKVEPKSC

[a] Disulfide-linked knob-into-hole mutations are highlighted in cyan. See Table 1 for the hinge region sequences.

Supplementary Table 2. Amino acid sequence of BA2[a]

Knob chain	Hole chain
HC<br>EVQLQQSGAELVKPGASVKLSCTPSGFNIKDTYMHWVKQRPEQGLEWIGR<br>IDPANGYTEYDPKFQDKATITADTSSNTAYLQLSSLTSEDTAVYYCADTQ<br>LYYWGQGTTLTVSSASTKGPSVFPLAPSSKSTSGGTAALGCLVKDYFPEP<br>VTVSWNSGALTSGVHTFPAVLQSSGLYSLSSVVTVPSSSLGTQTYICNVN<br>HKPSNTKVDKKVEPKS**---Hinge Region---**PAPELLGGPSVFLFPP<br>KPKDTLMISRTPEVTCVVVDVSHEDPEVKFNWYVDGVEVHNAKTKPREEQ<br>YNSTYRVVSVLTVLHQDWLNGKEYKCKVSNKALPAPIEKTISKAKGQPRE<br>PQVYTLPPCRDELTKNQVSLWCLVKGFYPSDIAVEWESNGQPENNYKTTP<br>PVLDSDGSFFLYSKLTVDKSRWQQGNVFSCSVMHEALHNHYTQKSLSLSP<br>GK	***HC***<br>EQVQLKESGPGLVAPSQSLSITCTVSGFSLTVYGVNWVRQPPGKGLEWLG<br>MIWGDGSTAYNSALKSRLTITKDNSKTQVFLKMNSLQTDDTARYYCARDG<br>RRYALDYWGQGTSVTVSSASVAAPSVFIFPPSDEQLKSGTASVVCLLNNF<br>YPREAKVQWKVDNALQSGNSQESVTEQDSKDSTYSLSSTLTLSKADYEKH<br>KVYACEVTHQGLSSPVTKSFNRGE**---Hinge region---**PAPELLGG<br>PSVFLFPPKPKDTLMISRTPEVTCVVVDVSHEDPEVKFNWYVDGVEVHNA<br>KTKPREEQYNSTYRVVSVLTVLHQDWLNGKEYKCKVSNKALPAPIEKTIS<br>KAKGQPREPQVCTLPPSRDELTKNQVSLSCAVKGFYPSDIAVEWESNGQP<br>ENNYKTTPPVLDSDGSFFLVSKLTVDKSRWQQGNVFSCSVMHEALHNHYT<br>QKSLSLSPGK
LC<br>QIVLTQSPAIMSASLGERVTMTCTASSSVSSTYLHWYQQKPGSSPKLWIY<br>STSNLASGVPARFSGSGSGTSYSLTISNMEAEDAATYYCHQYHRSPLTFG<br>AGTKLELKRTVAAPSVFIFPPSDEQLKSGTASVVCLLNNFYPREAKVQWK<br>VDNALQSGNSQESVTEQDSKDSTYSLSSTLTLSKADYEKHKVYACEVTHQ<br>GLSSPVTKSFNRGEC	***LC***<br>DIVLTQSPTSLAVSLGQRATISCRASESVDSYGDSFLHWYQQKPGQPPIL<br>LIYRASNLDSGIPARFSGSGSRTDFTLTINPVEADDVATYYCQQSNEDPY<br>TFGGGTKVTVLSSASTKGPSVFPLAPSSKSTSGGTAALGCLVKDYFPEPV<br>TVSWNSGALTSGVHTFPAVLQSSGLYSLSSVVTVPSSSLGTQTYICNVNH<br>KPSNTKVDKKVEPKSC

[a] Disulfide-linked knob-into-hole mutations are highlighted in cyan. See Table 1 for the hinge region sequences.

Supplementary Table 3. Amino acid sequence of BA3[a]

Knob chain	Hole chain
HC<br>KVQLQQSGAELVKPGASVKLSCKASGYTFTESIIHWVKQRSGQGLEWIGW<br>FYPGSDNINYNEKFKDKATLTADKSSSTVYMELTRLTSEDSAVYFCASHE<br>GPYVYFDYWGQGTTLTVSSASTKGPSVFPLAPSSKSTSGGTAALGCLVKD<br>YFPEPVTVSWNSGALTSGVHTFPAVLQSSGLYSLSSVVTVPSSSLGTQTY<br>ICNVNHKPSNTKVDKKVEPKS**---Hinge region---**PAPELLGGPSV<br>FLFPPKPKDTLMISRTPEVTCVVVDVSHEDPEVKFNWYVDGVEVHNAKTK<br>PREEQYNSTYRVVSVLTVLHQDWLNGKEYKCKVSNKALPAPIEKTISKAK<br>GQPREPQVYTLPPCRDELTKNQVSLWCLVKGFYPSDIAVEWESNGQPENN<br>YKTTPPVLDSDGSFFLYSKLTVDKSRWQQGNVFSCSVMHEALHNHYTQKS<br>LSLSPGK	***HC***<br>QVQLKESGPGLVAPSQSLSITCTVSGFSLTVYGVNWVRQPPGKGLEWLGM<br>IWGDGSTAYNSALKSRLTITKDNSKTQVFLKMNSLQTDDTARYYCARDGR<br>RYALDYWGQGTSVTVSSASVAAPSVFIFPPSDEQLKSGTASVVCLLNNFY<br>PREAKVQWKVDNALQSGNSQESVTEQDSKDSTYSLSSTLTLSKADYEKHK<br>VYACEVTHQGLSSPVTKSFNRGE**---Hinge region---**PAPELLGGP<br>SVFLFPPKPKDTLMISRTPEVTCVVVDVSHEDPEVKFNWYVDGVEVHNAK<br>TKPREEQYNSTYRVVSVLTVLHQDWLNGKEYKCKVSNKALPAPIEKTISK<br>AKGQPREPQVCTLPPSRDELTKNQVSLSCAVKGFYPSDIAVEWESNGQPE<br>NNYKTTPPVLDSDGSFFLVSKLTVDKSRWQQGNVFSCSVMHEALHNHYTQ<br>KSLSLSPGK
LC<br>DIVMTQSHKFMSTSVGDRVSITCKASQDVSTAVAWYQQKPGQSPKLLIYW<br>TSTRHTGVPDRFTGSGSGTDYTLTISSVQAEDLALYYCQHHYSTPYTFGG<br>GTKLEIQRTVAAPSVFIFPPSDEQLKSGTASVVCLLNNFYPREAKVQWKV<br>DNALQSGNSQESVTEQDSKDSTYSLSSTLTLSKADYEKHKVYACEVTHQG<br>LSSPVTKSFNRGEC	***LC***<br>DIVLTQSPTSLAVSLGQRATISCRASESVDSYGDSFLHWYQQKPGQPPIL<br>LIYRASNLDSGIPARFSGSGSRTDFTLTINPVEADDVATYYCQQSNEDPY<br>TFGGGTKVTVLSSASTKGPSVFPLAPSSKSTSGGTAALGCLVKDYFPEPV<br>TVSWNSGALTSGVHTFPAVLQSSGLYSLSSVVTVPSSSLGTQTYICNVNH<br>KPSNTKVDKKVEPKSC

[a] Disulfide-linked knob-into-hole mutations are highlighted in cyan. See Table 1 for the hinge region sequences.

Supplementary Table 4. Cryo-EM data collection and processing

Dataset	1:1 complex of BA1-GP4 and TNFR2
EMDB accession code	EMD-63050
PDB accession code	9LFL
Magnification	60,000
Voltage (kV)	300
Electron exposure ($e^-/Å^2$)	80
Defocus range (μm)	−0.5 to −2.0
Pixel size (Å)	1.048
Symmetry imposed	C1
Imported movies (no.)	5,508
Initial particle images (no.)	2,971,529
Final particle images (no.)	178,242
Map resolution (Å)	3.73
FSC threshold	0.143

Supplementary Table 5. Eigenvalues and the contribution of the 1st–5th principal components (PC1-5)

PC	value	contribution
1	101.023	65.7%
2	25.4846	16.6%
3	18.4627	12.0%
4	4.56841	3.0%
5	1.03969	0.7%

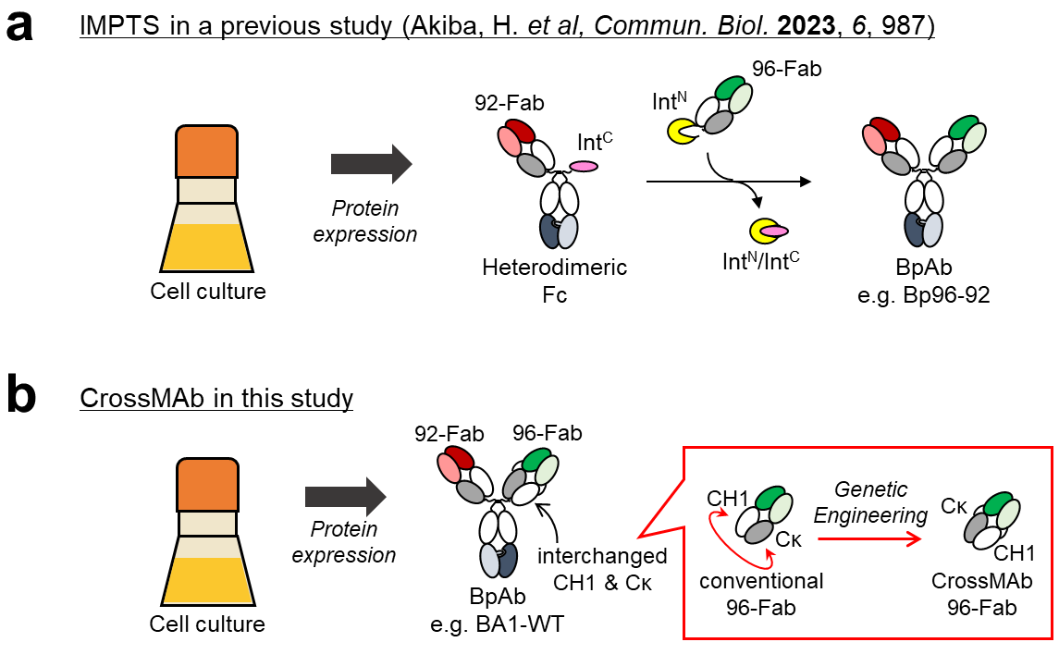

Supplementary Figure 1. Format transformation from intein-mediated protein trans-splicing (IMPTS) into CrossMAb.

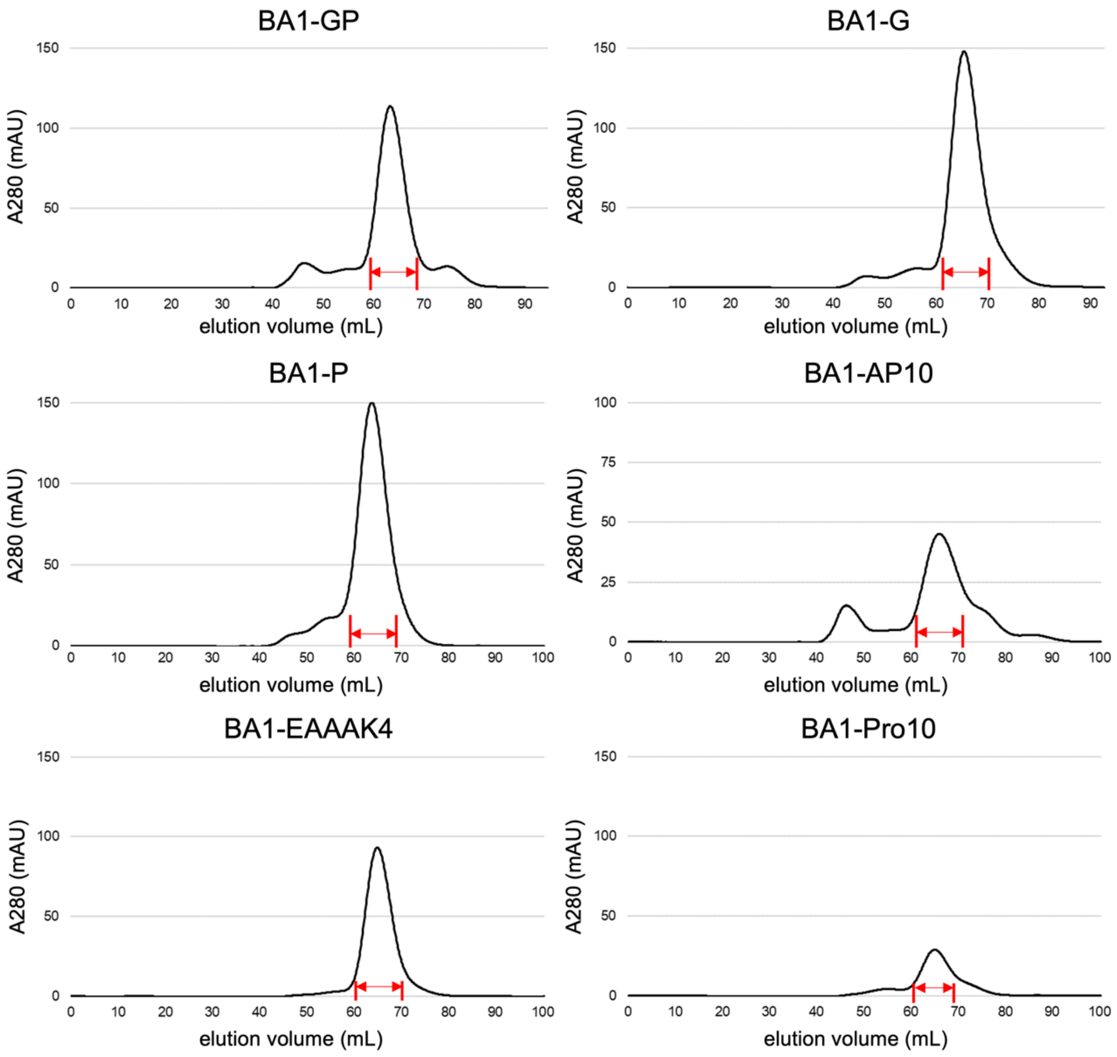

Supplementary Figure 2. Preparative size-exclusion chromatograms of BA1 inserted with a peptide linker into one of heavy chains. The fractions with red arrows were collected.

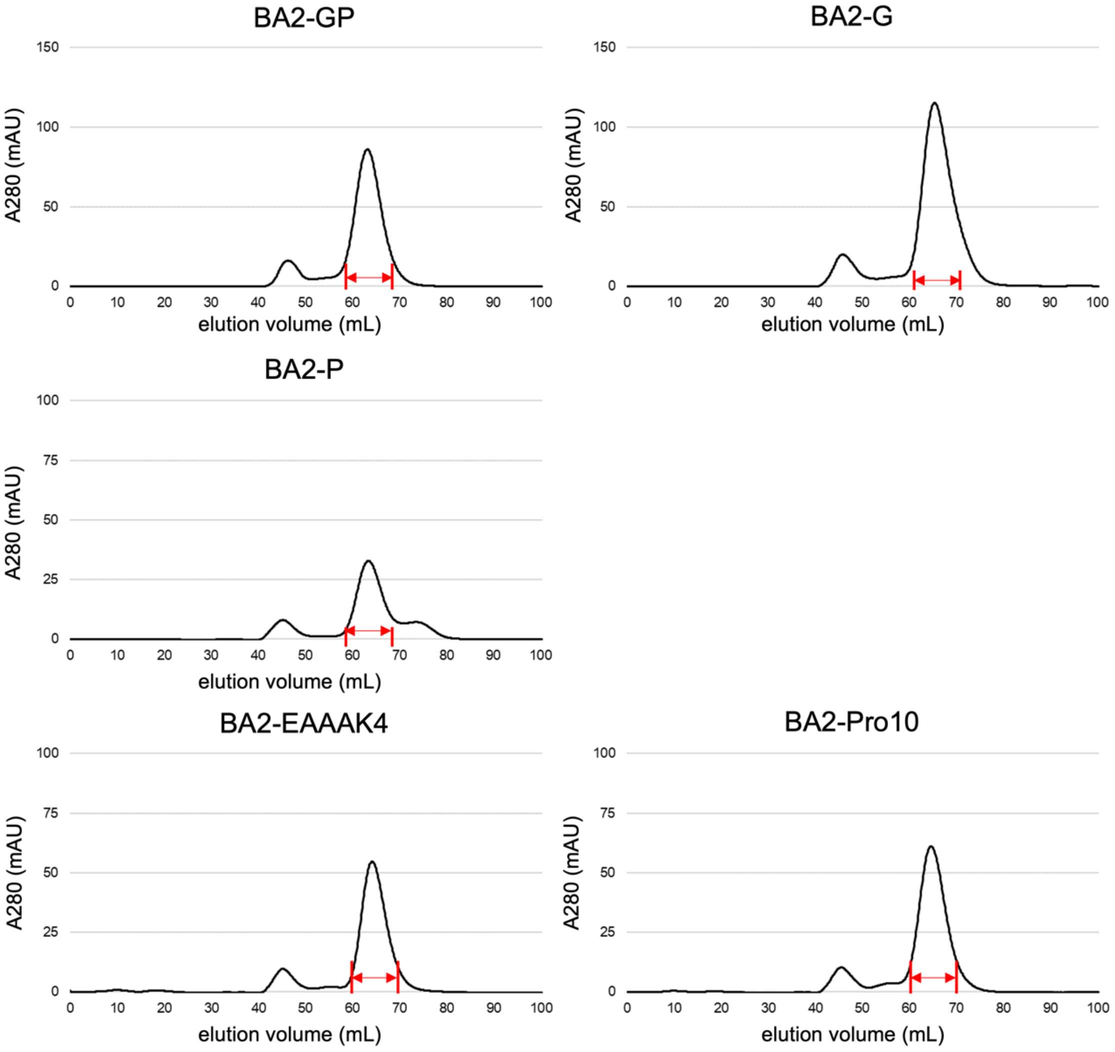

Supplementary Figure 3. Preparative size-exclusion chromatograms of BA2 inserted with a peptide linker into one of heavy chains. The fractions with red arrows were collected.

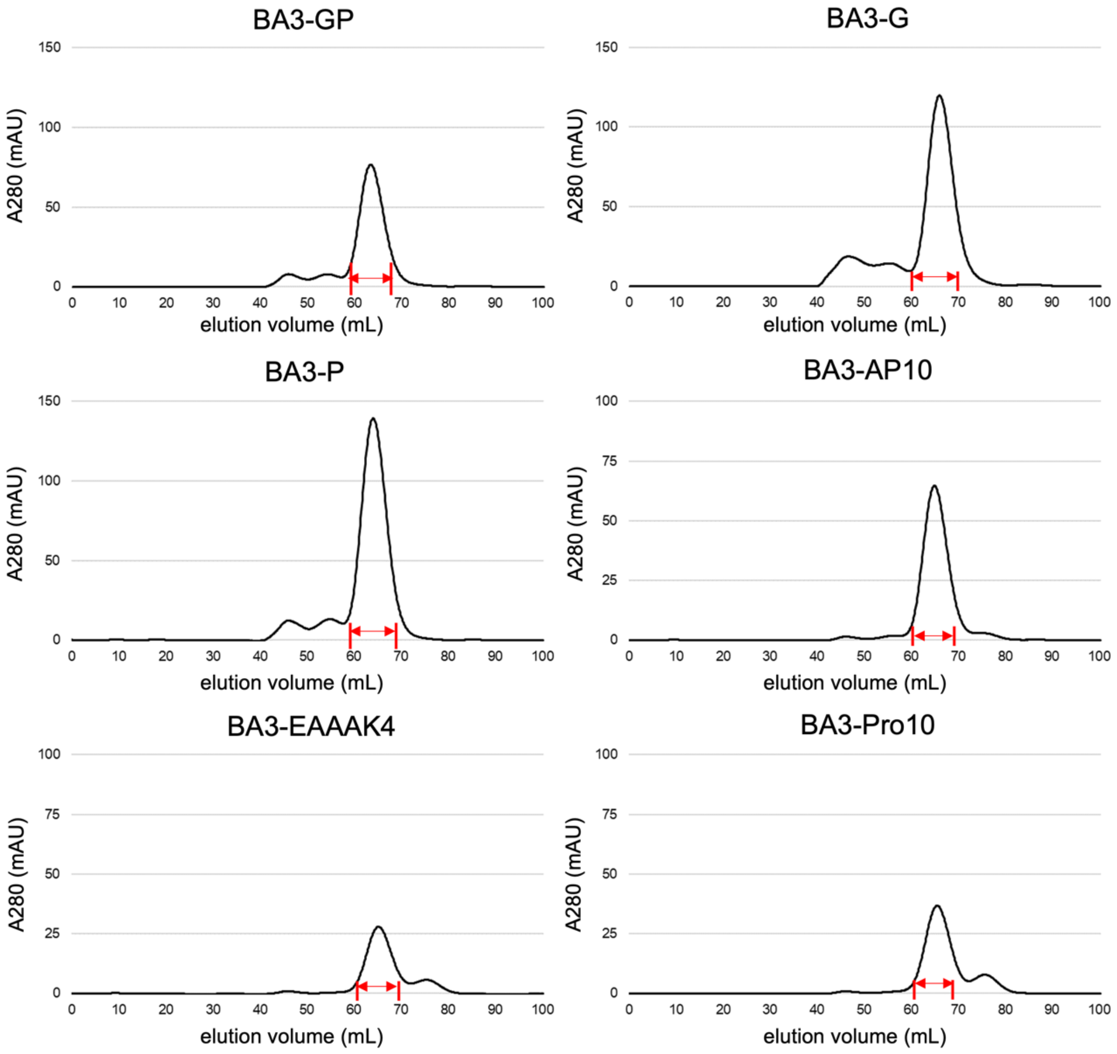

Supplementary Figure 4. Preparative size-exclusion chromatograms of BA3 inserted with a peptide linker into one of heavy chains. The fractions with red arrows were collected.

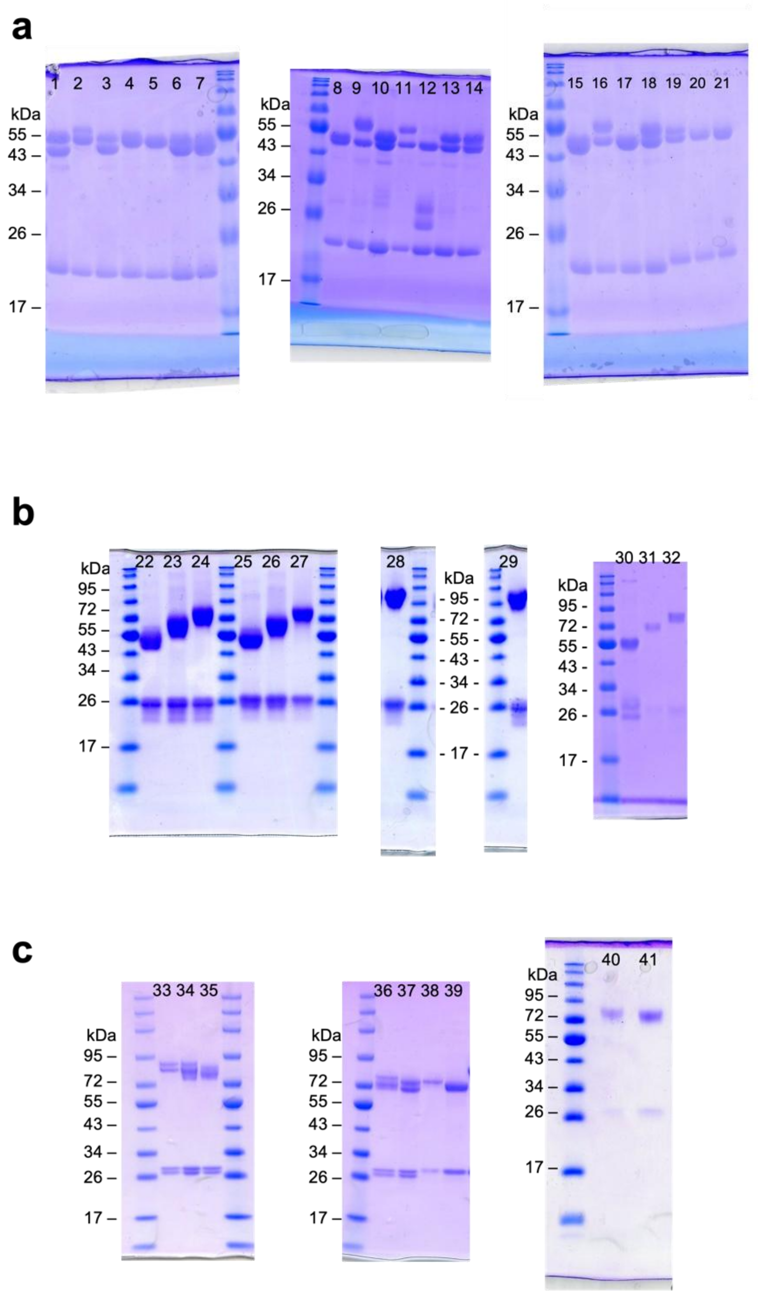

Lane No.	Protein
1	BA1-WT
2	BA1-GP
3	BA1-G
4	BA1-P
5	BA1-AP10
6	BA1-EAAAK4
7	BA1-Pro10
8	BA2-WT
9	BA2-GP
10	BA2-G
11	BA2-P
12	BA2-AP10
13	BA2-EAAAK4
14	BA2-Pro10
15	BA3-WT
16	BA3-GP
17	BA3-G
18	BA3-P
19	BA3-AP10
20	BA3-EAAAK4
21	BA3-Pro10
22	BA1-WT
23	BA1-GP2
24	BA1-GP4
25	BA2-WT
26	BA2-GP2
27	BA2-GP4
28	BA1-GP8
29	BA2-GP8
30	BA3-WT
31	BA3-GP2
32	BA3-GP4
33	BA1- GP4 (+gly)
34	BA1-GP4 (+gly and –gly)
35	BA1-GP4 (-gly)
36	BA1-GP2 (+gly)
37	BA1-GP2 (-gly)
38	BA2-GP2 (+gly)
39	BA2-GP2 (-gly)
40	BA1-GP4 (+gly)
41	BA1-GP4 (-gly)

Supplementary Figure 5. SDS-PAGE analysis of BpAbs with a peptide linker into one of heavy chains (**a**), with multiple GP linkers into both of heavy chains (**b**), before (+gly) and after (-gly) deglycosylation (**c**).

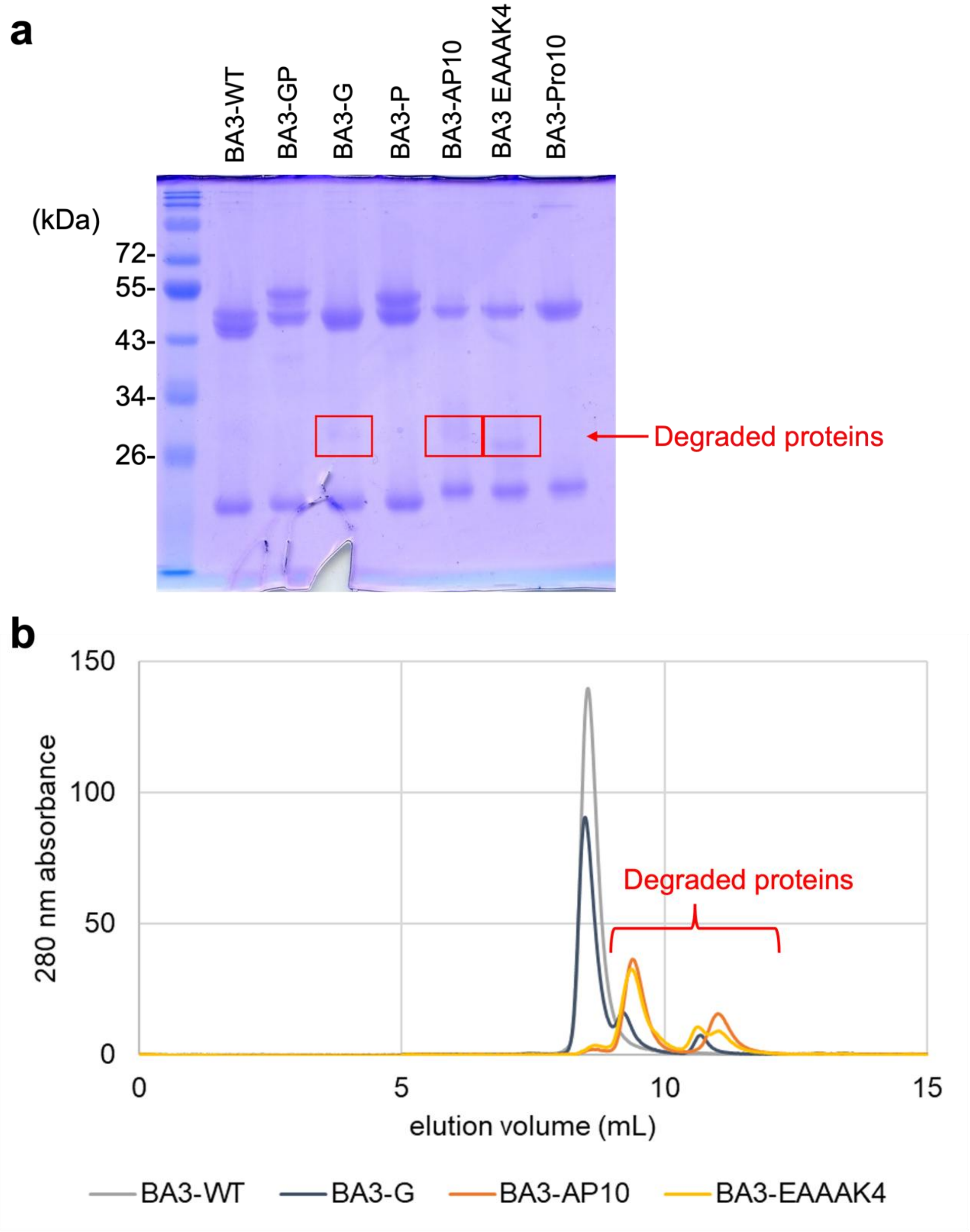

Supplementary Figure 6. Degradation of biparatopic antibodies stored at 4 °C, one month after purification. Analysis with SDS-PAGE (**a**) and with TSKgel G3000SWXL size-exclusion column (**b**).

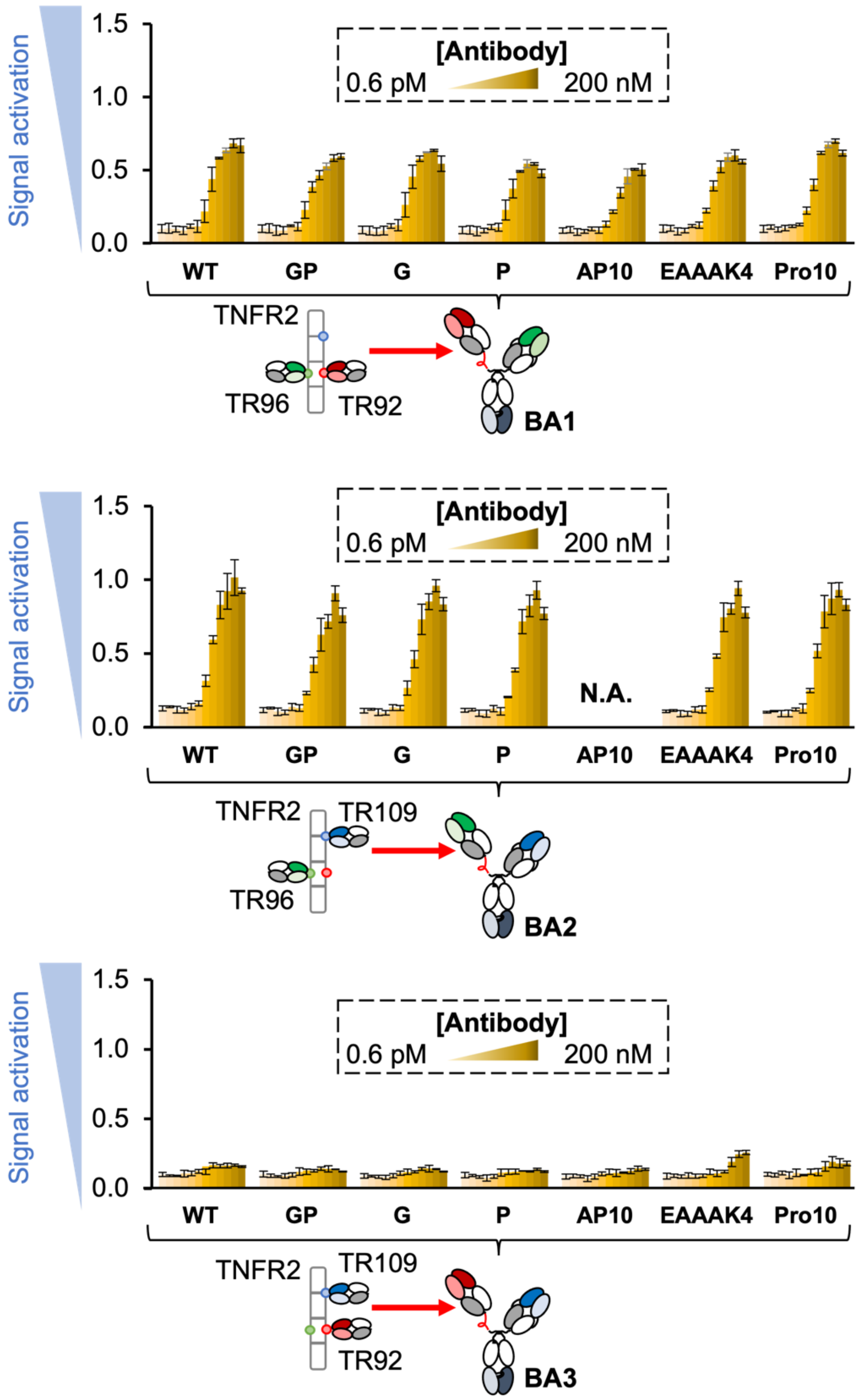

Supplementary Figure 7. Control of biological activities through linker insertion into the hinge region of one of heavy chains. Agonistic activities of BpAbs in a reporter gene assay are shown. BA2-AP10 was not analyzed due to degradation after purification. Values are shown with the standard deviation of three independent experiments.

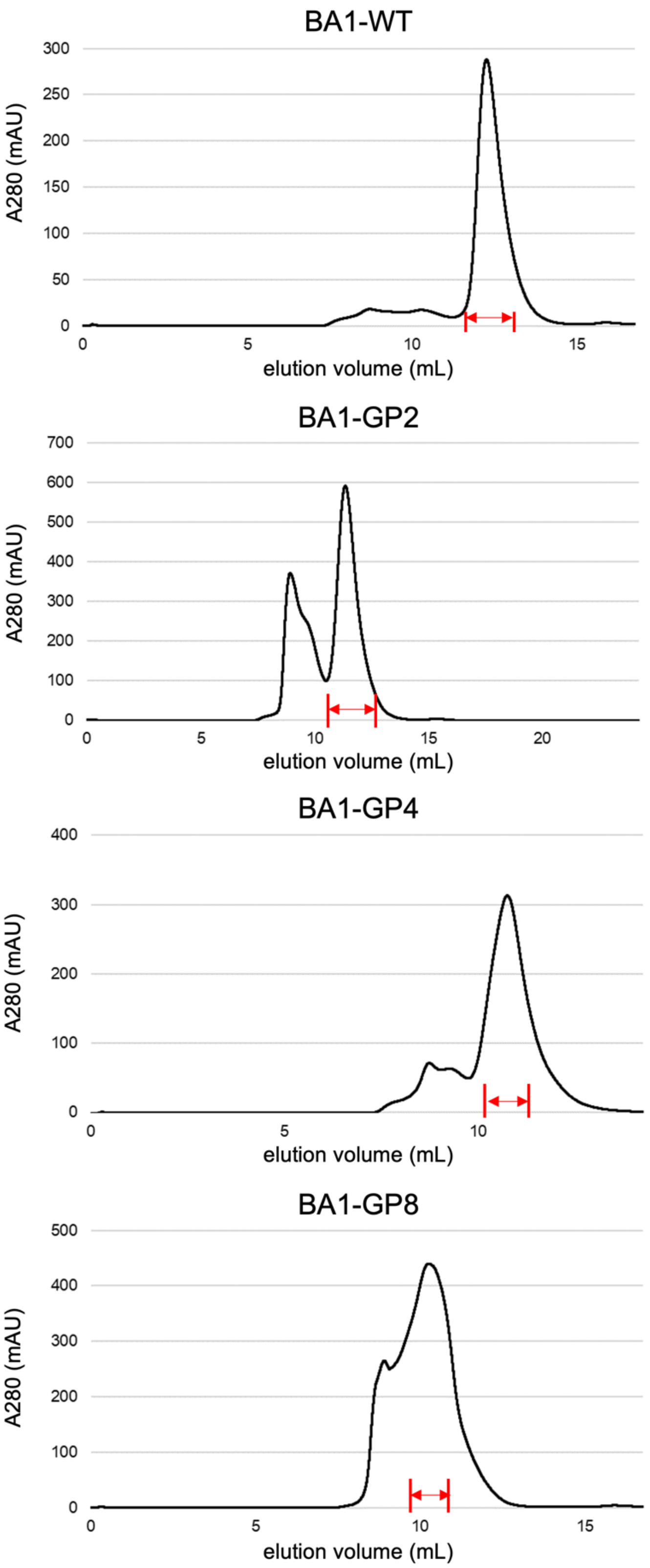

Supplementary Figure 8. Preparative size-exclusion chromatograms of BA1-WT, BA1-GP2, BA1-GP4 and BA1-GP8. The fractions with red arrows were collected.

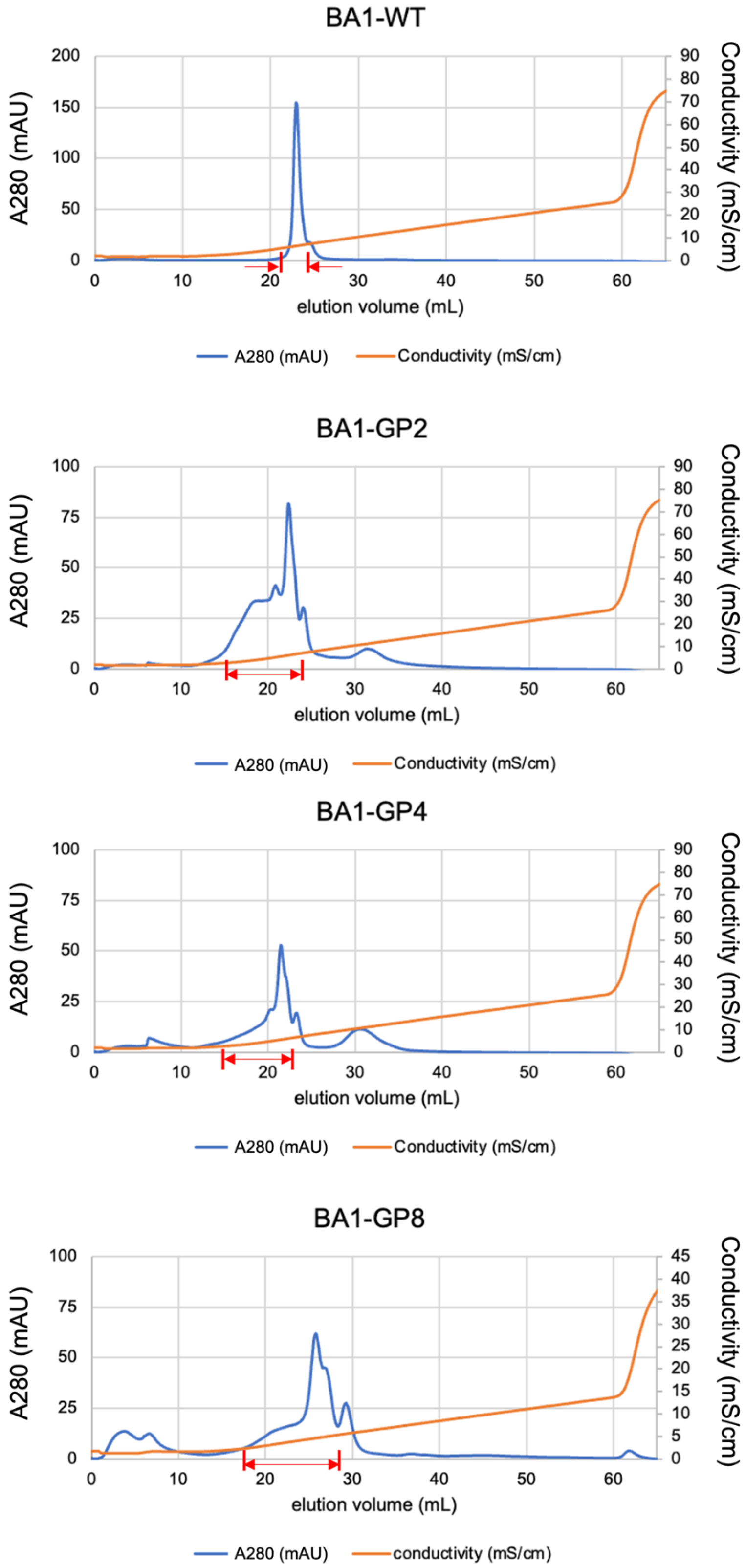

Supplementary Figure 9. Preparative cation-exchange chromatograms of BA1-WT, BA1-GP2, BA1-GP4 and BA1-GP8. The fractions with red arrows were collected.

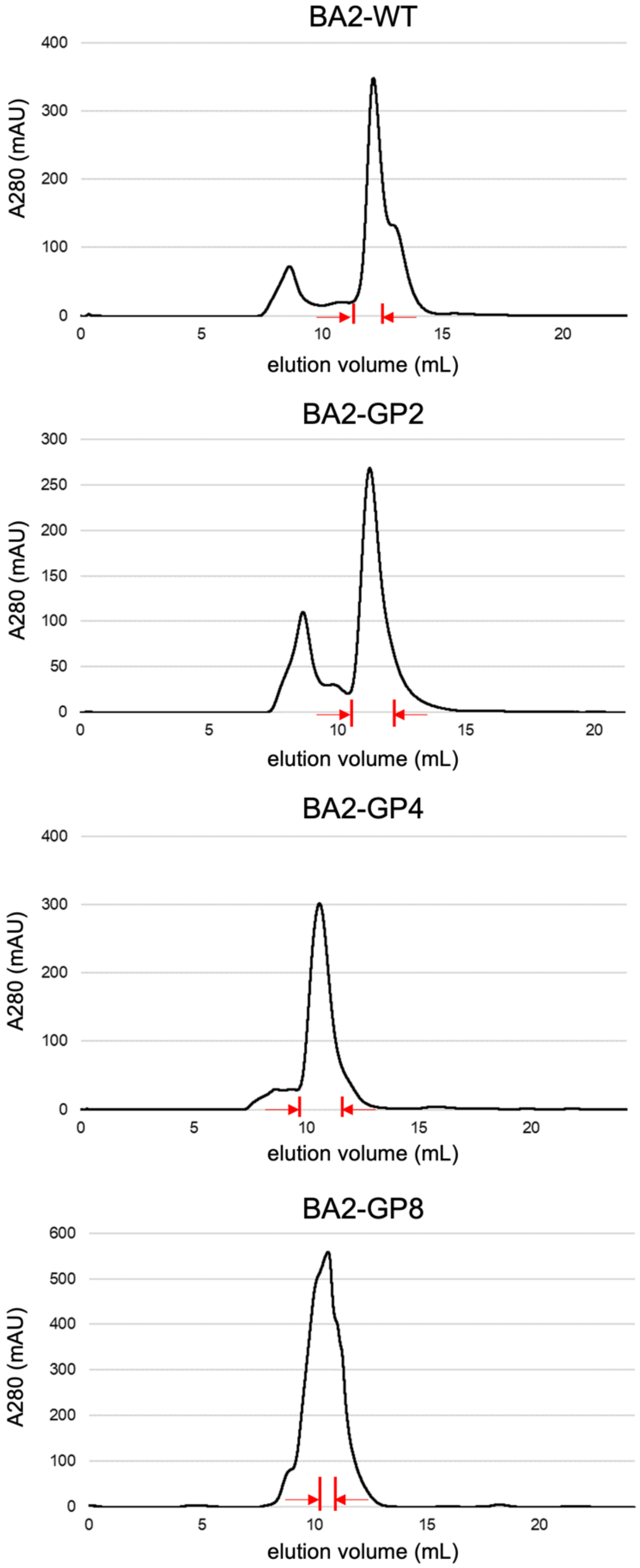

Supplementary Figure 10. Preparative size-exclusion chromatograms of BA2-WT, BA2-GP2, BA2-GP4 and BA2-GP8. The fractions with red arrows were collected.

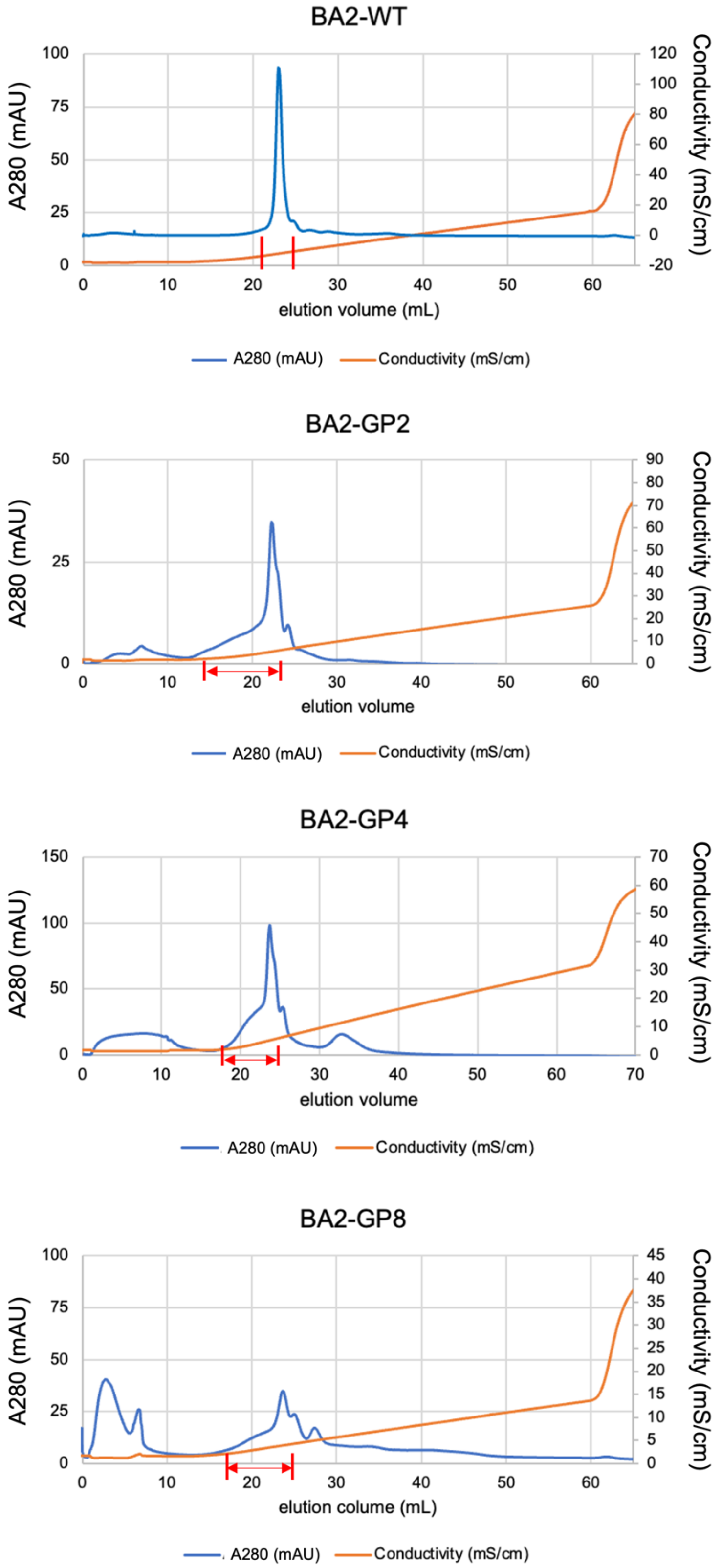

Supplementary Figure 11. Preparative cation-exchange chromatograms of BA2-WT, BA2-GP2, BA2-GP4 and BA2-GP8. The fractions with red arrows were collected.

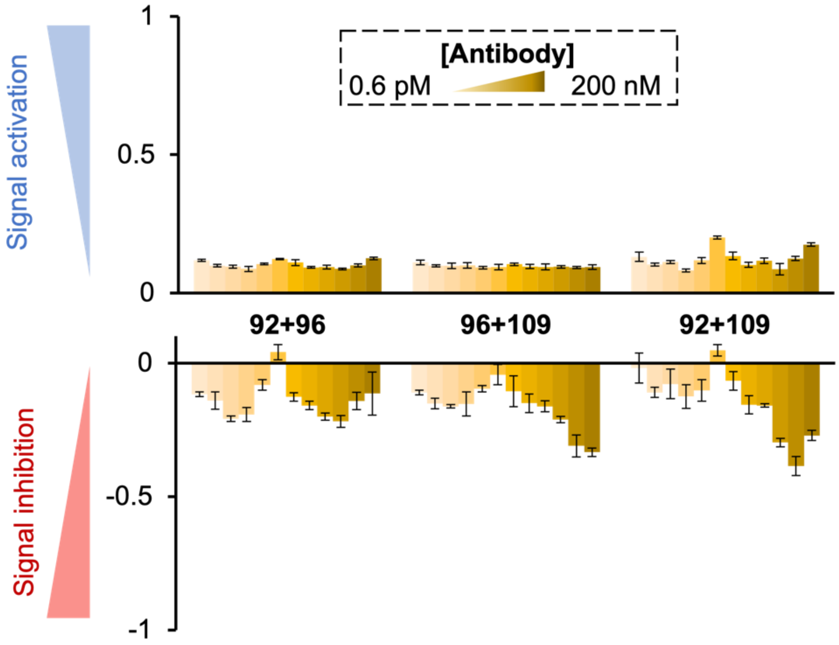

Supplementary Figure 12. Agonistic and antagonistic activities of the mixtures of two Fab proteins in a reporter gene assay. Values are shown with the standard deviation of three independent experiments.

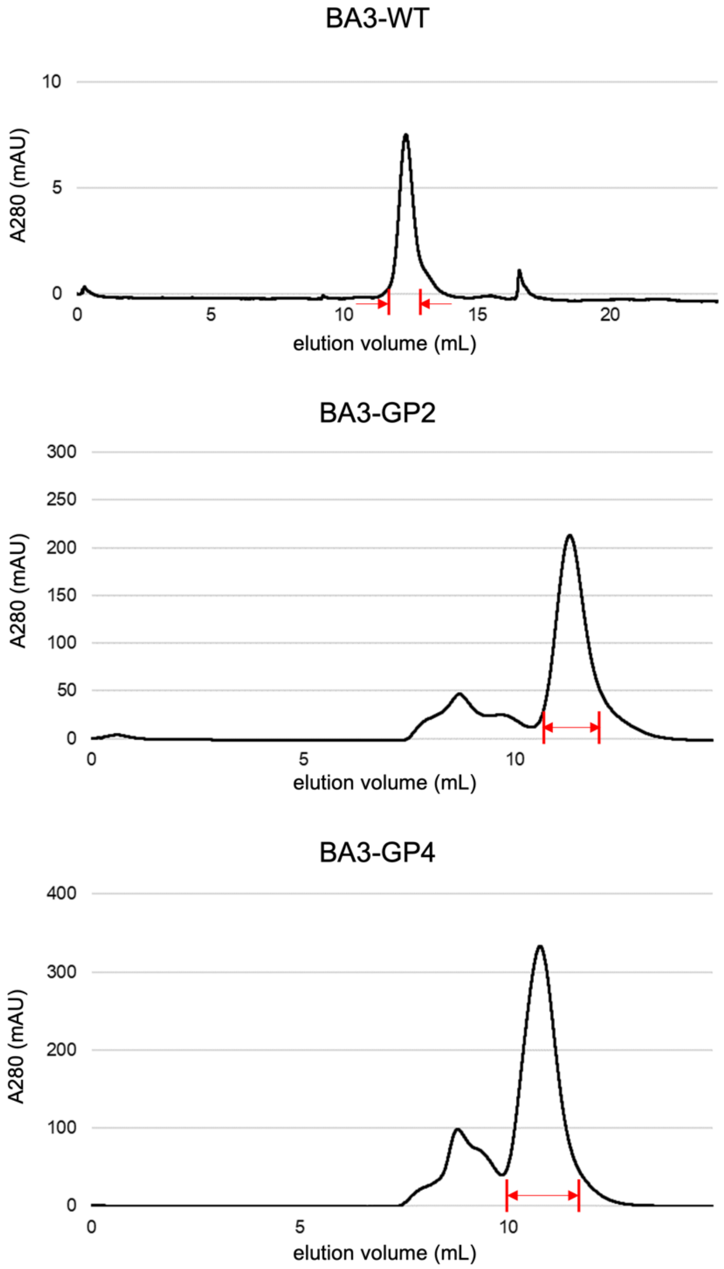

Supplementary Figure 13. Preparative size-exclusion chromatograms of BA3-WT, BA3-GP2 and BA3-GP4. The fractions with red arrows were collected.

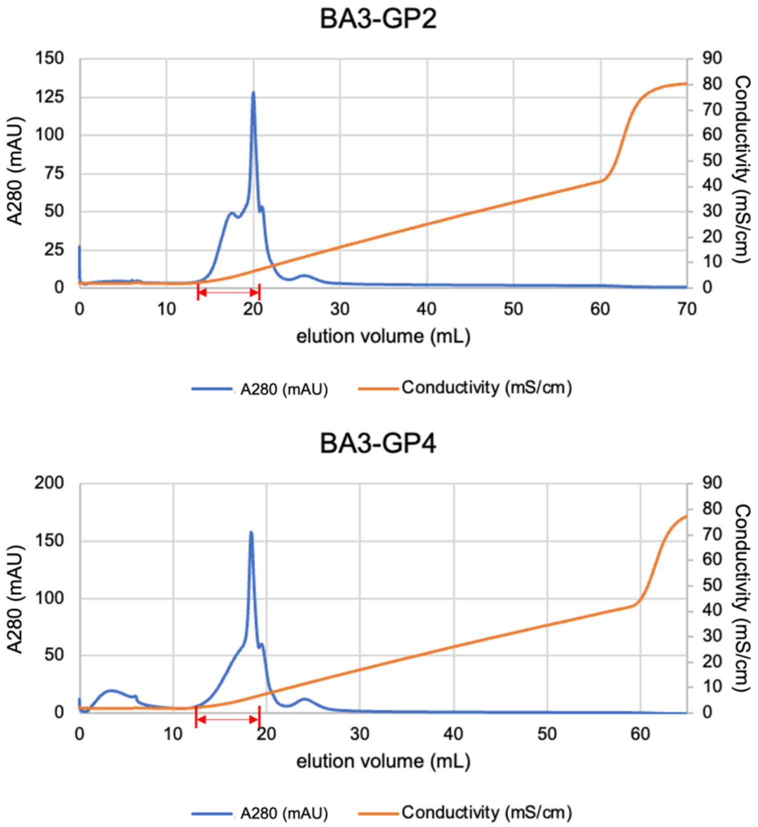

Supplementary Figure 14. Preparative cation-exchange chromatograms of BA3-GP2 and BA3-GP4. The fractions with red arrows were collected.

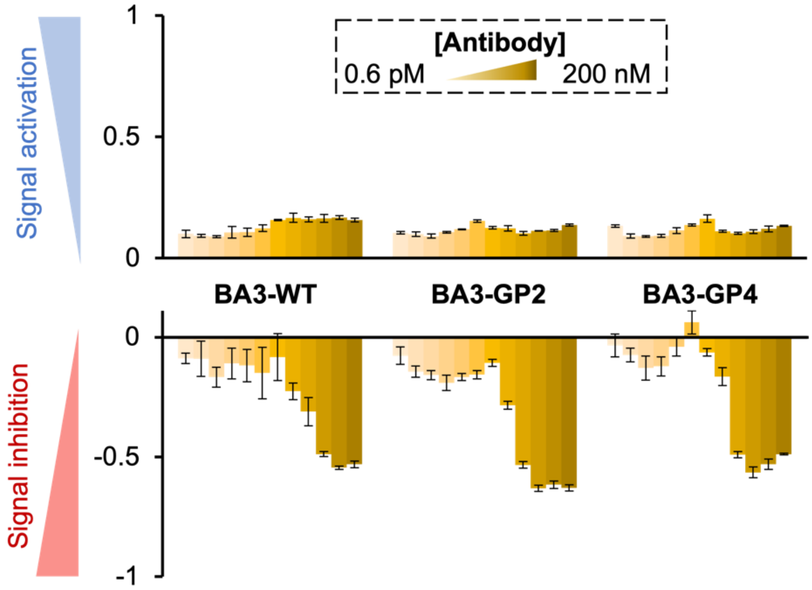

Supplementary Figure 15. Agonistic (upper) and antagonistic (lower) activities of BA3-WT, BA3 GP2 and BA3-GP4 in a reporter gene assay. Values are shown with the standard deviation of three independent experiments.

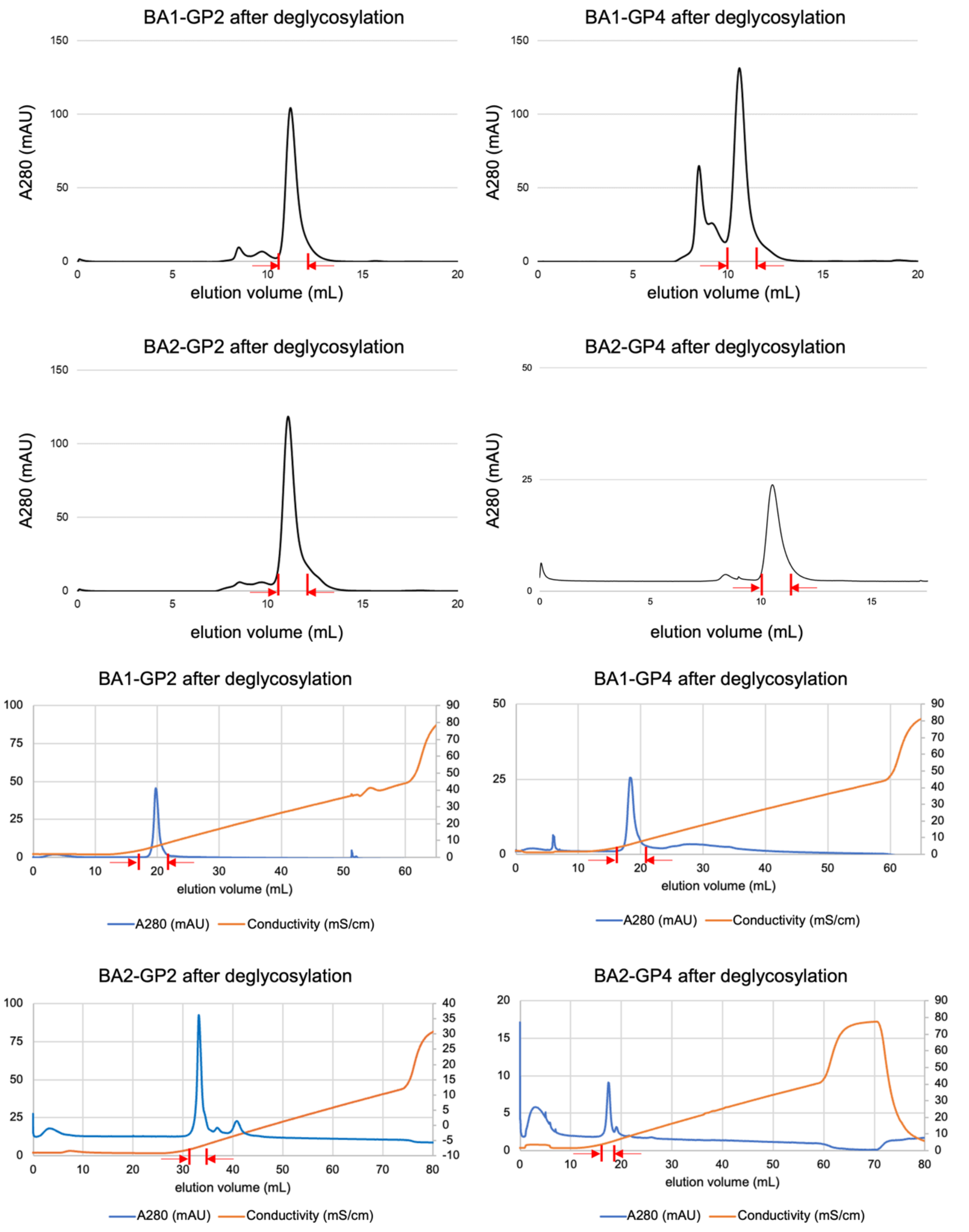

Supplementary Figure 16. Preparative size-exclusion chromatograms (upper four charts) and cation-exchange chromatograms (lower four charts) of BA1-GP2, BA1-GP4, BA2-GP2 and BA2-GP4 after deglycosylation. The fractions with red arrows were collected.

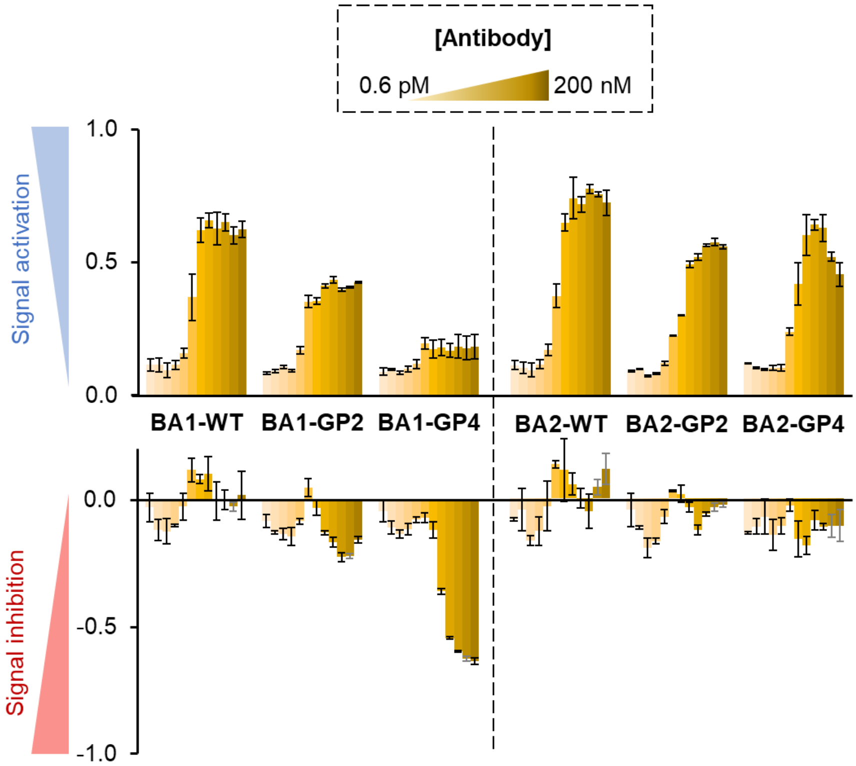

Supplementary Figure 17. Agonistic (upper) and antagonistic (lower) activities of BpAbs after deglycosylation in a reporter gene assay. Values are shown with the standard deviation of three independent experiments.

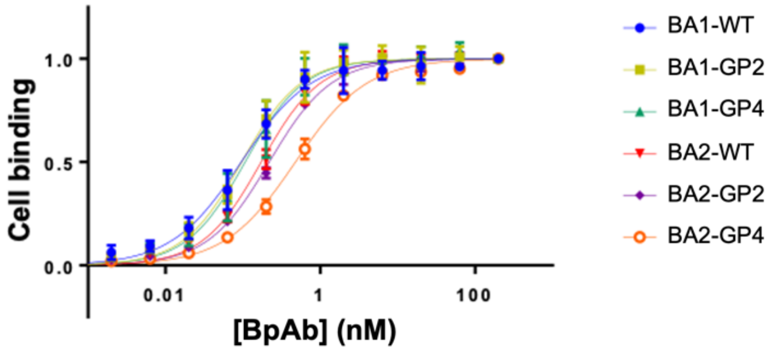

	BA1-WT	BA1-GP2	BA1-GP4	BA2-WT	BA2-GP2	BA2-GP4
**log [EC50/nM]**	-1.021±0.041	-1.015±0.035	-0.971±0.038	-0.763±0.026	-0.659±0.017	-0.321±0.018
**EC50 (nM)**	0.095	0.097	0.107	0.173	0.219	0.478

Supplementary Figure 18. Apparent binding affinity of BpAbs to TNFR2-expressing Ramos-Blue cells analyzed by using flow cytometry.

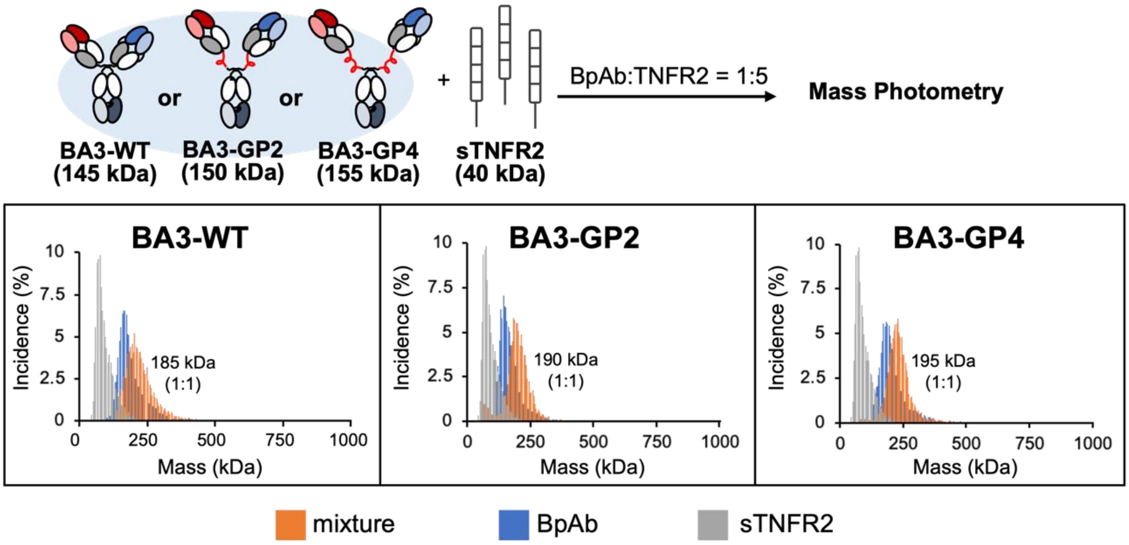

Supplementary Figure 19. Immunocomplex formed between BA3 and TNFR2. BpAbs were mixed with 5 eq. of sTNFR2.

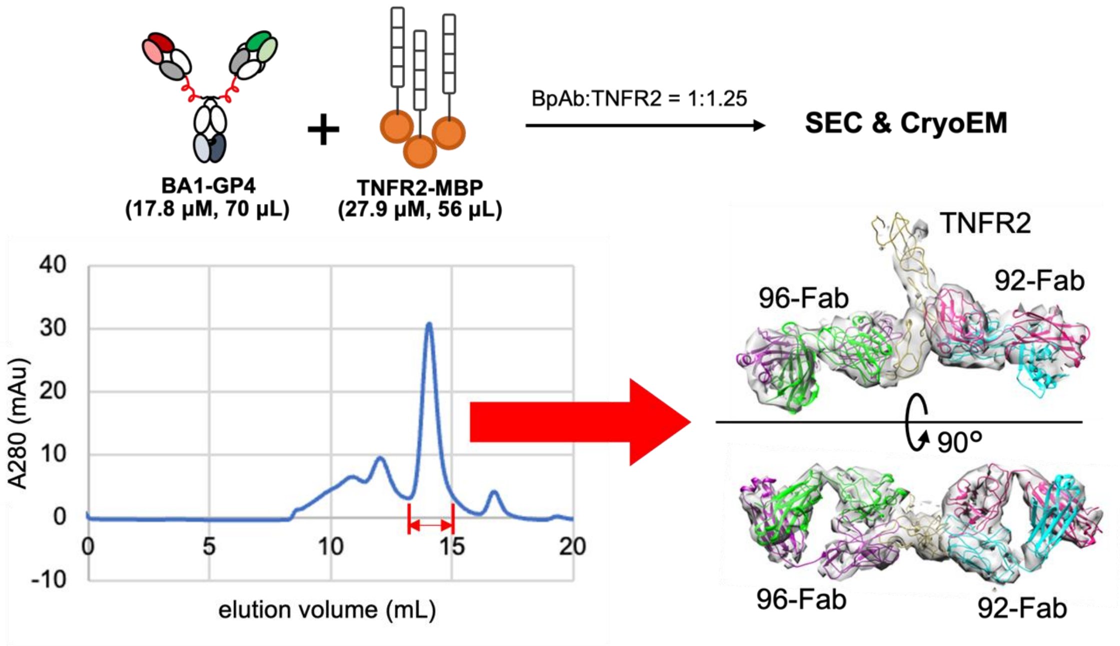

Supplementary Figure 20. Sample preparation for cryo-electron microscopy. Immunocomplex was collected by size-exclusion chromatography for cryo-electron microscopy.

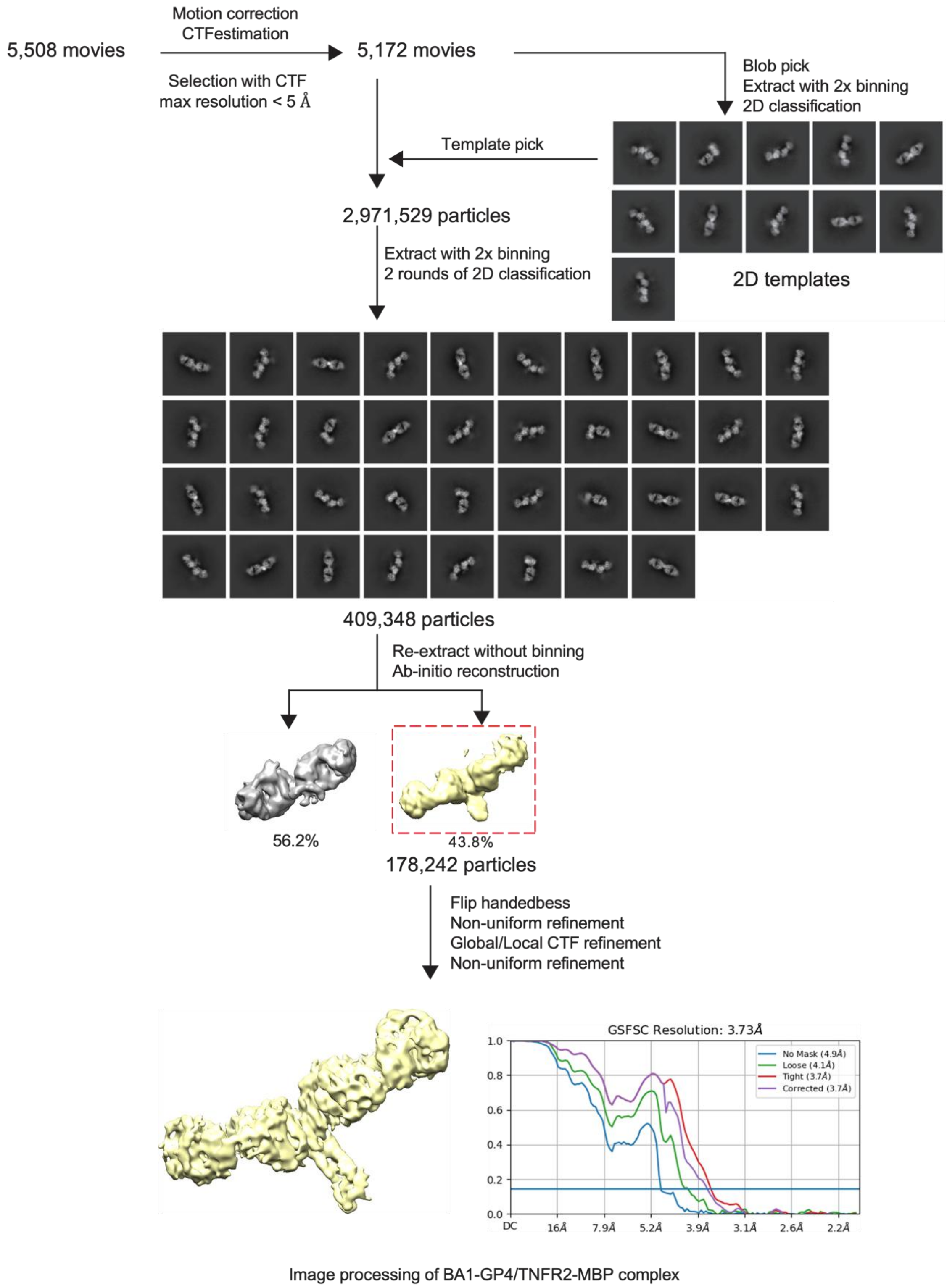

Supplementary Figure 21. Image processing of BA1-GP4/TNFR2-MBP complex.

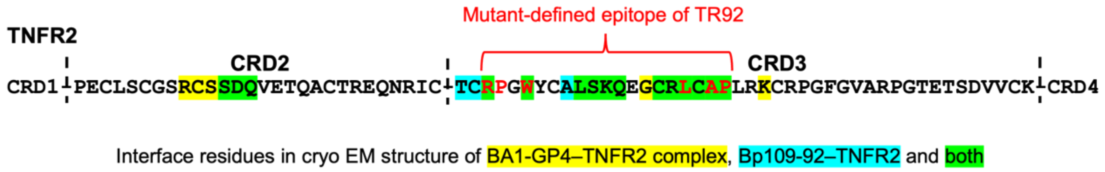

Supplementary Figure 22. Amino-acid residues of TNFR2 in the interface with 92-Fab from two different BpAbs. Interface residues are defined as Δ(buried surface area) > 0 calculated in PISA server. Interface residues that overlap in BA1-GP4–TNFR2 and Bp109-92–TNFR2 complexes are highlighted in green. Interface residues found only for BA1-GP4–TNFR2 or Bp109-92–TNFR2 are highlighted in yellow or cyan, respectively. Red texts indicate the core epitope residues defined by mutagenesis in the previous report (Akiba, H. *et al. Commun. Biol.* **6**, 987 (2023)).

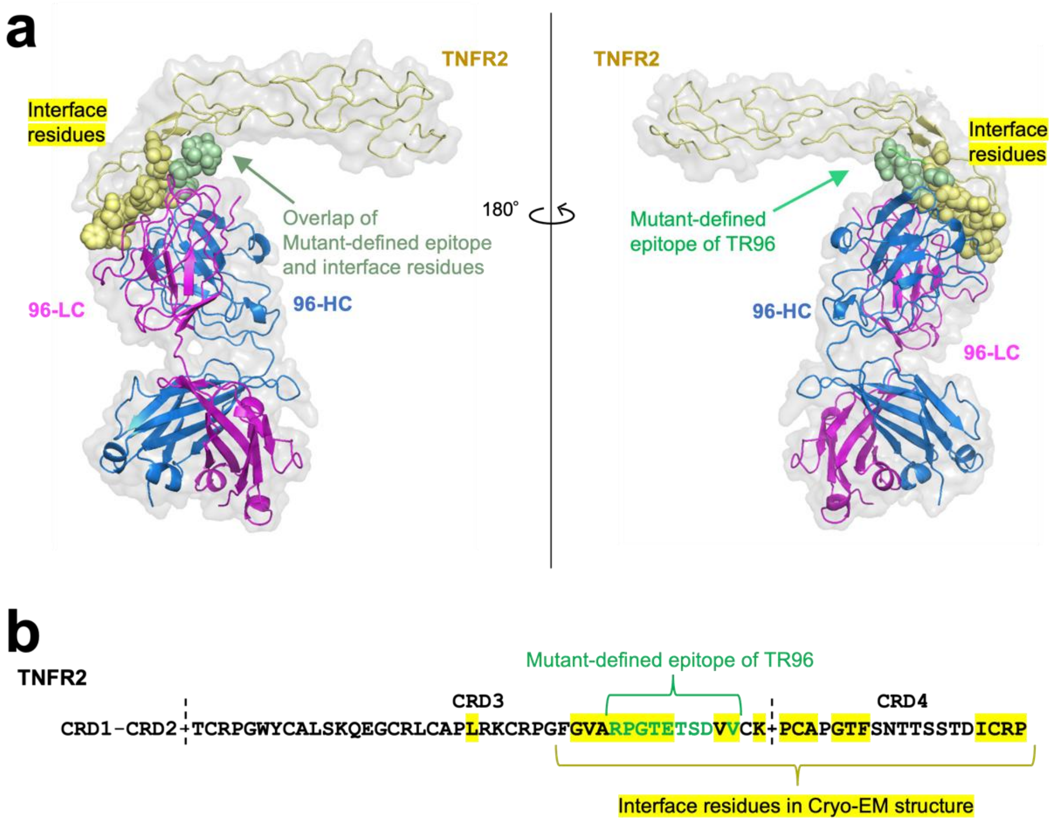

Supplementary Figure 23. The epitope of 96-Fab binding to TNFR2. (**a**) The epitope mapped on the complex structure of 96-Fab and TNFR2 from PDB ID 9LFL. Amino-acid residues of TNFR2 in the interface with 96-Fab are shown with spheres. Interface residues are defined as Δ(buried surface area) > 0 calculated in PISA server. TNFR2, 96-HC and 96-LC are respectively shown in yellow, blue and magenta. Epitope region found by mutagenesis in a previous study (Akiba, H. *et al. Commun. Biol.* **6**, 987 (2023)) are colored green. Because the mutagenesis was conducted by loop-level substitution, amino acid residues without direct contact are also included in this region. (**b**) Highlighted amino acid residues in (**a**).

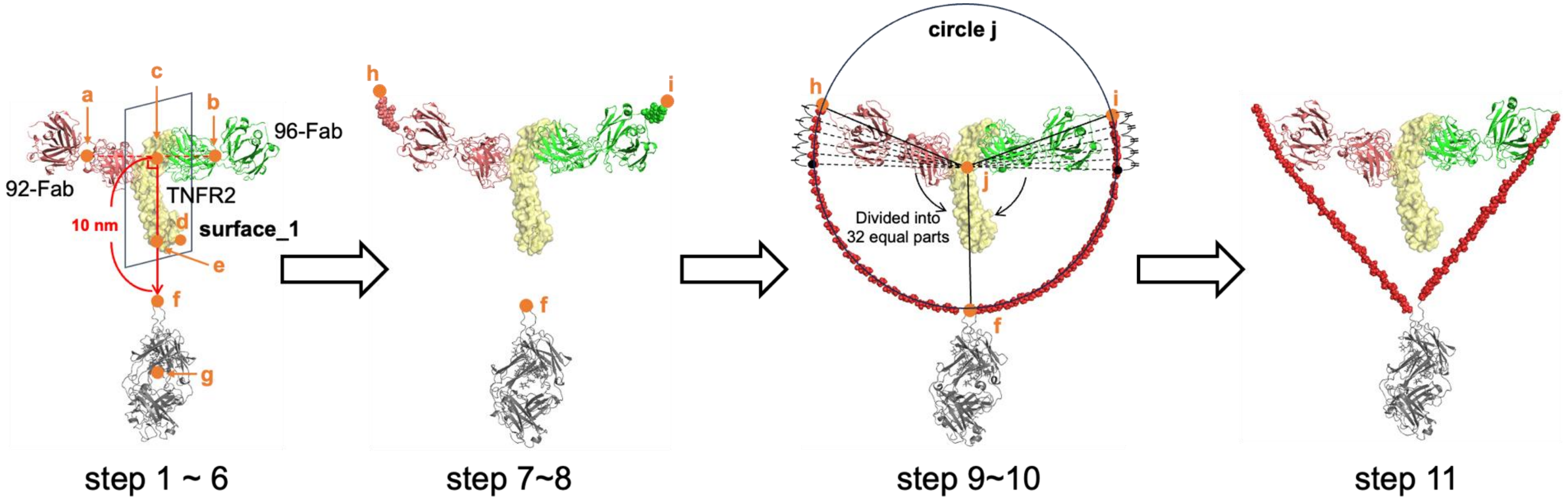

1. Centroids of all atoms in 92-Fab and 96-Fab are defined as **a** and **b**, respectively
2. **Surface_1** was defined as a plane that passes through the midpoint (**c**) of points **a** and **b**, with the line **ab** as the normal
3. Point **e** on the surface_1 was defined as the closest point to the N-terminal amino acid residue of TNFR2 (**d**)
4. Point **f** was defined on the line **ce**, located 10 nm away from **c**
5. N-terminal disulfide bond of Fc was positioned on **f**
6. Centroid of all amino acids in the Fc was defined as point **g** and positioned on the line **ce**
7. Missing C-terminal amino acid residues of two Fabs were inserted
8. C-termini of the heavy chains of 92-Fab and 96-Fab were defined as points **h** and **i**, respectively
9. A circle that passes through points **f**, **h**, and **i** was defined as circle **j**
10. Amino acid residues of the linkers were positioned at an equal interval along the circle **j**
11. Energy minimization was performed in MOE

Supplementary Figure 24. Strategy for building the initial structure of the complexes of BA1-GP2 and TNFR2.

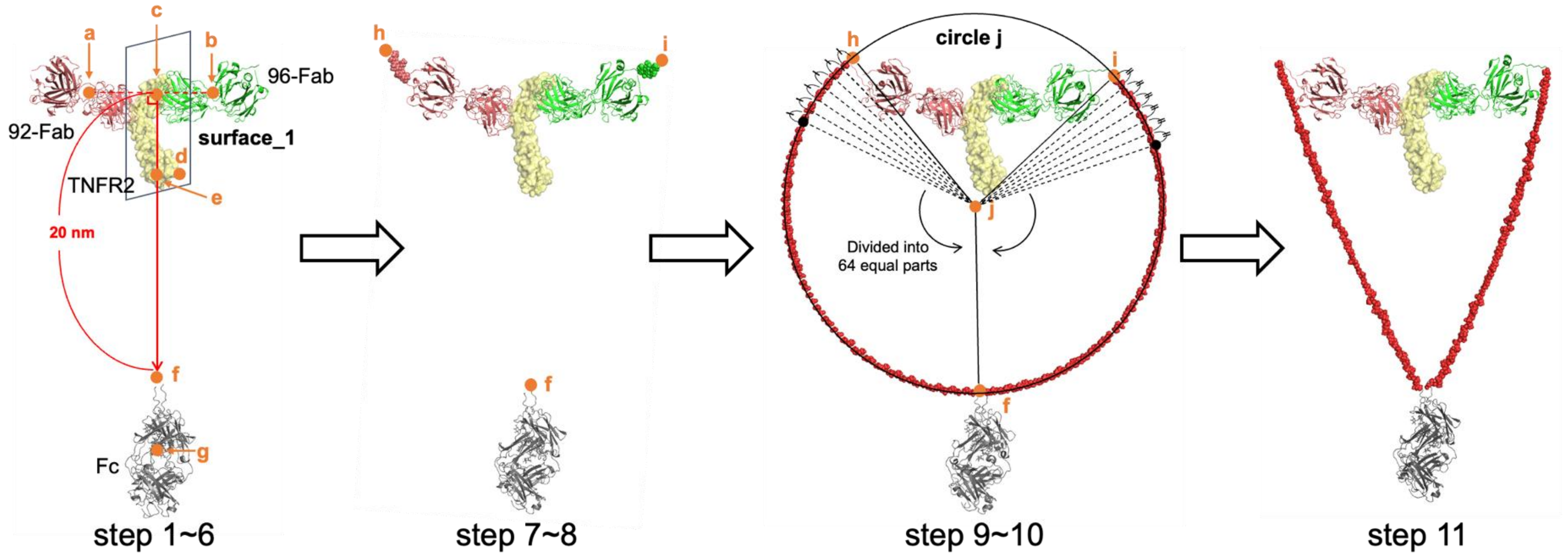

1. Centroids of all atoms in 92-Fab and 96-Fab are defined as **a** and **b**, respectively
2. **Surface_1** was defined as a plane that passes through the midpoint (**c**) of points **a** and **b**, with the line **ab** as the normal
3. Point **e** on the surface_1 was defined as the closest point to the N-terminal amino acid residue of TNFR2 (**d**)
4. Point **f** was defined on the line **ce**, located 20 nm away from **c**
5. N-terminal disulfide bond of Fc was positioned on **f**
6. Centroid of all amino acids in the Fc was defined as point **g** and positioned on the line **ce**
7. Missing C-terminal amino acid residues of two Fabs were inserted
8. C-termini of the heavy chains of 92-Fab and 96-Fab were defined as points **h** and **i**, respectively
9. A circle that passes through points **f**, **h**, and **i** was defined as circle **j**
10. Amino acid residues of the linkers were positioned at an equal interval along the circle **j**
11. Energy minimization was performed in MOE

Supplementary Figure 25. Strategy for building the initial structure of the complexes of BA1-GP4 and TNFR2.

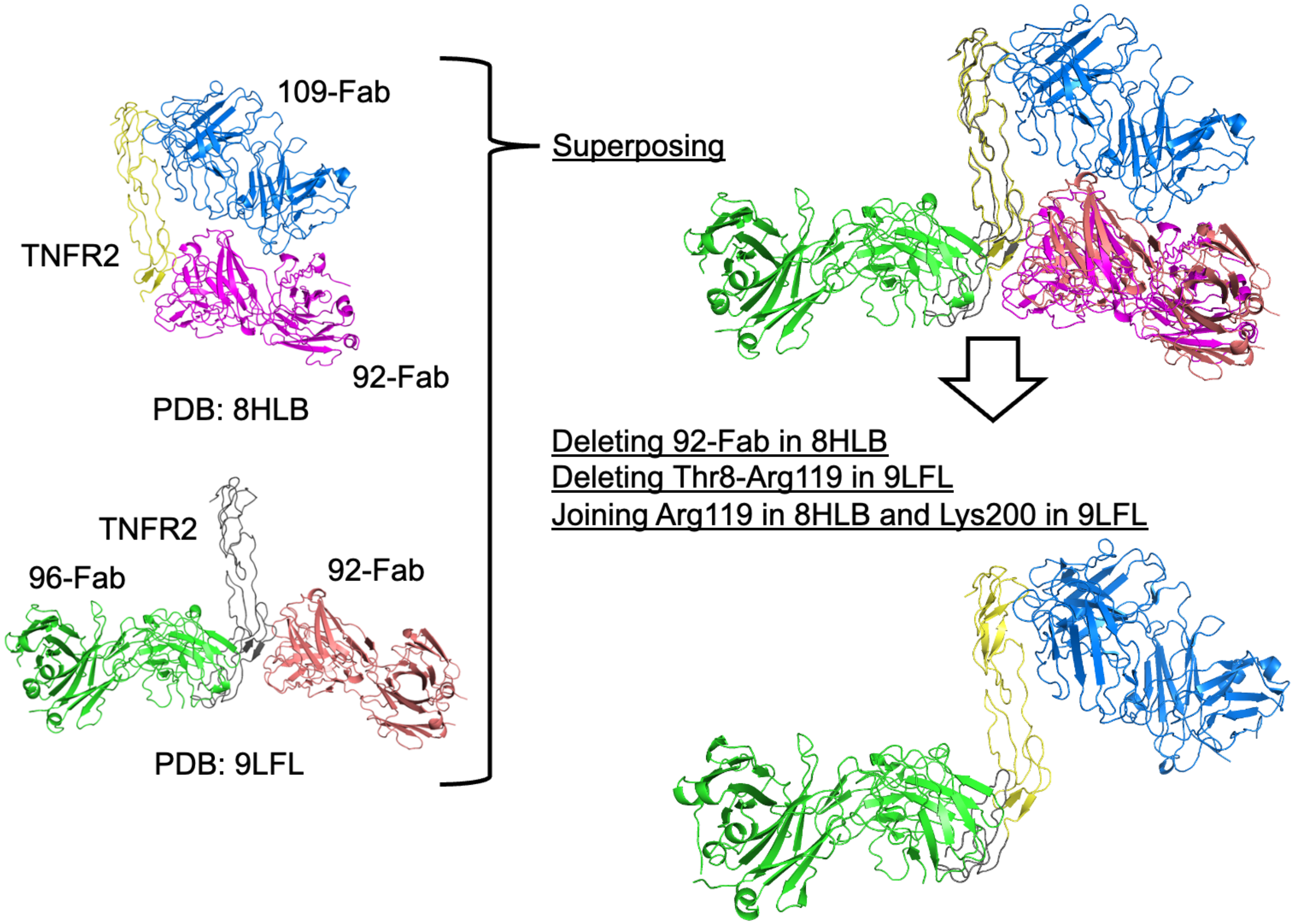

Supplementary Figure 26. Strategy for building ternary complex of 109-Fab, 96-Fab and TNFR2.

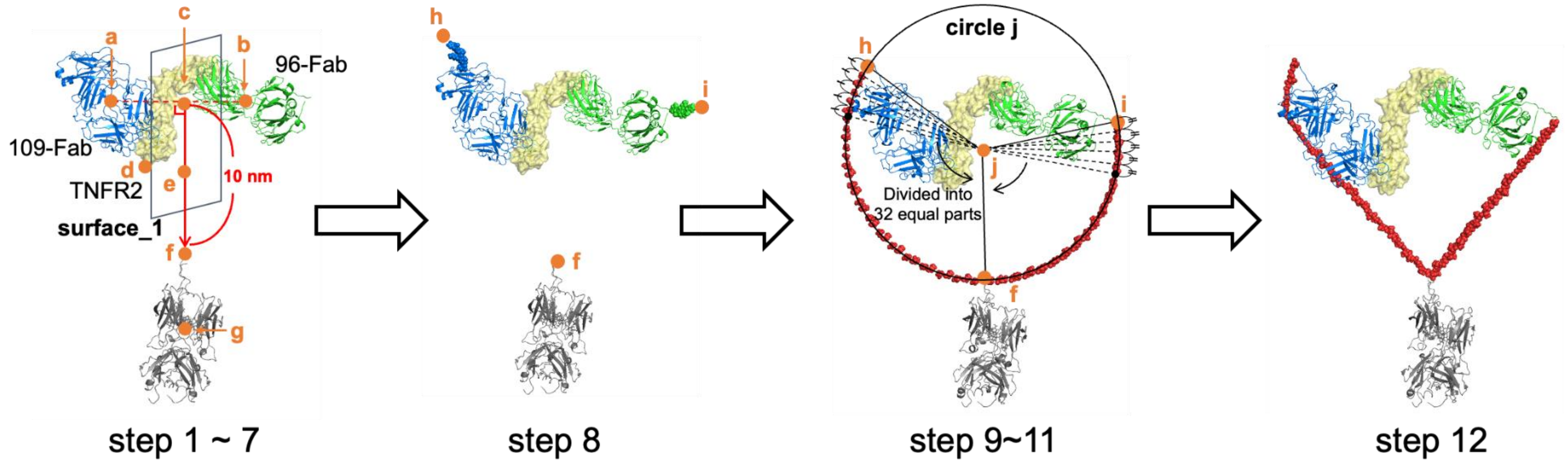

1. Centroids of all atoms in 109-Fab and 96-Fab are defined as **a** and **b**, respectively
2. **Surface_1** was defined as a plane that passes through the midpoint (**c**) of points **a** and **b**, with the line **ab** as the normal
3. Point **e** on the surface_1 was defined as the closest point to the N-terminal amino acid residue of TNFR2 (**d**)
4. Point **f** was defined on the line **ce**, located 10 nm away from **c**
5. N-terminal disulfide bond of Fc was positioned on **f**
6. Centroid of all amino acids in the Fc was defined as point **g** and positioned on the line **ce**
7. Missing C-terminal amino acid residues of two Fabs were inserted
8. C-termini of the heavy chains of 109-Fab and 96-Fab were defined as points **h** and **i**, respectively
9. A circle that passes through points **f**, **h**, and **i** was defined as circle **j**
10. Amino acid residues of the linkers were positioned at an equal interval along the circle **j**
11. Energy minimization was performed in MOE

Supplementary Figure 27. Strategy for building the initial structure of the complexes of BA2-GP2 and TNFR2.

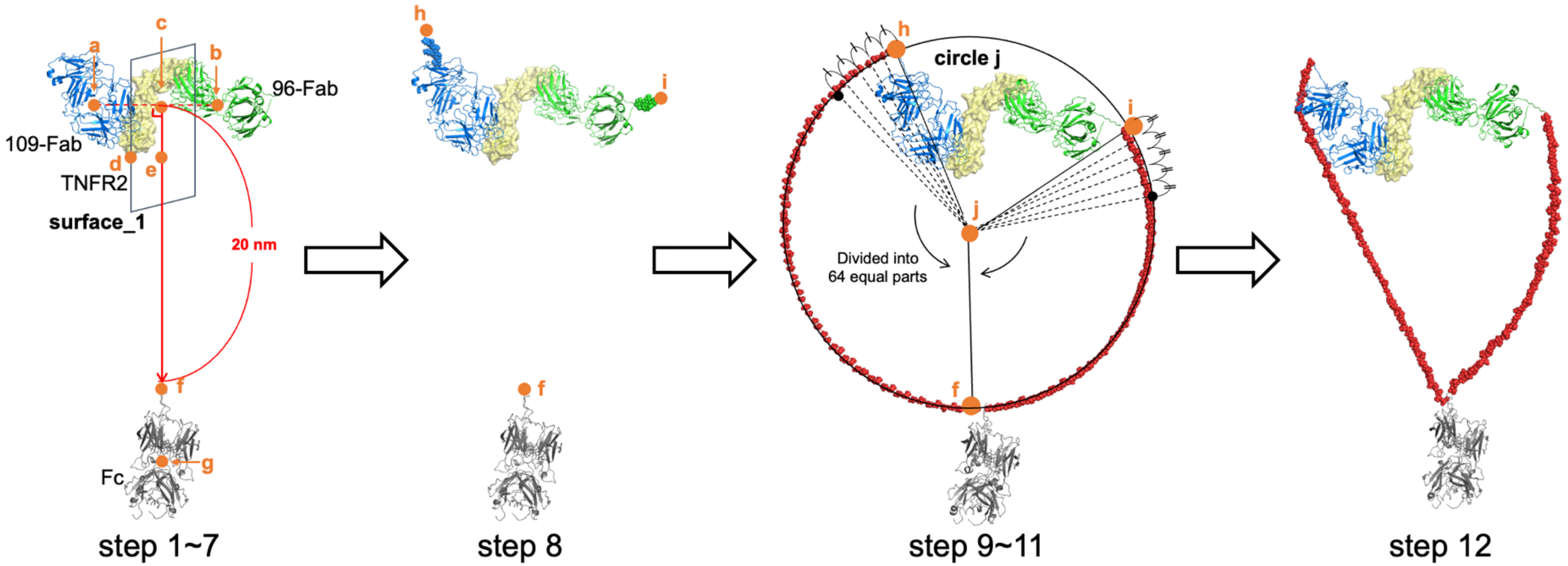

1. Centroids of all atoms in 109-Fab and 96-Fab are defined as **a** and **b**, respectively
2. **Surface_1** was defined as a plane that passes through the midpoint (**c**) of points **a** and **b**, with the line **ab** as the normal
3. Point **e** on the surface_1 was defined as the closest point to the N-terminal amino acid residue of TNFR2 (**d**)
4. Point **f** was defined on the line **ce**, located 20 nm away from **c**
5. N-terminal disulfide bond of Fc was positioned on **f**
6. Centroid of all amino acids in the Fc was defined as point **g** and positioned on the line **ce**
7. Missing C-terminal amino acid residues of two Fabs were inserted
8. C-termini of the heavy chains of 109-Fab and 96-Fab were defined as points **h** and **i**, respectively
9. A circle that passes through points **f**, **h**, and **i** was defined as circle **j**
10. Amino acid residues of the linkers were positioned at an equal interval along the circle **j**
11. Energy minimization was performed in MOE

Supplementary Figure 28. Strategy for building the initial structure of the complexes of BA2-GP4 and TNFR2.
